# Palladium nanoparticles immobilized on DT-CH-modified MgFe_2_O_4_@APTES magnetic nanoparticles as an efficient and reusable new catalyst for C-C coupling reactions

**DOI:** 10.1038/s41598-025-25753-3

**Published:** 2025-11-25

**Authors:** Magda Abdel lattif H, Mohammad Abu Shuheil, Munthar Abosaoda, M. M. Rekha, Rasha Eldalawy, Subhashree Ray, Kattela Chennakesavulu, Renu Sharma

**Affiliations:** 1 Centre for Research Impact & Outcome, Chitkara University Institute of Engineering and Technology, Chitkara University, Rajpura, 140401 Punjab India; 2 Department of Medical Laboratory Sciences, Faculty of Allied Medical Sciences, Hourani Center for Applied Scientific Research, Al-Ahliyya Amman University, Amman, Jordan; 3 College of Pharmacy, The Islamic University, Najaf, Iraq; 4 College of Pharmacy, The Islamic University of Al Diwaniyah, Al Diwaniyah, Iraq; 5 Department of Chemistry and Biochemistry, School of Sciences, JAIN (Deemed to be University), Bangalore, Karnataka India; 6 College of Pharmacy, Al-Turath University, Al Mansour, Baghdad, 10013 Iraq; 7 Department of Biochemistry, IMS and SUM Hospital, Siksha ’O’ Anusandhan (Deemed to be University), Bhubaneswar, 751003 Odisha India; 8 Department of Chemistry, Sathyabama Institute of Science and Technology, Chennai, Tamil Nadu India; 9 Department of Chemistry, University Institute of Sciences, Chandigarh University, Mohali, Punjab India

**Keywords:** Magnetic nanocomposite, MgFe_2_O_4_, Suzuki reaction, DTPA, Coupling reaction, Chemistry, Materials science

## Abstract

A novel protocol has been developed for synthesizing a DT-CH-Pd complex anchored on MgFe_2_O_4_ nanoparticles, referred to as MgFe_2_O_4_@APTES@DT-CH-Pd. The structure of this nanomagnetic catalyst was thoroughly analyzed using various techniques, including FT-IR, XRD, FE-SEM, EDX, ICP, TGA, VSM, and N₂ adsorption-desorption isotherm analysis. Subsequently, its catalytic performance was evaluated in Suzuki cross-coupling reactions conducted in water, highlighting its use as a green solvent. Palladium loading on the catalyst was quantified through ICP analysis, measuring at 1.7 × 10⁻³ mol g⁻¹. This methodology offers several advantages, such as straightforward preparation from readily available materials, excellent catalytic activity, ease of operation, short reaction times, high yields, and an environmentally friendly solvent system. Importantly, the nanocatalyst was conveniently recovered using an external magnet and reused multiple times without notable degradation in catalytic efficiency.

**Supplementary Information:**

The online version contains supplementary material available at 10.1038/s41598-025-25753-3.

## Introduction

Nanomaterials have gained substantial interest over the past decade owing to their unique chemical and catalytic characteristics^1–4^. One key area of application is their use as a substrate for immobilizing homogeneous catalysts, combining the benefits of both homogeneous and heterogeneous systems^5,6^. Within this realm, spinel metal oxides like ferrites have been extensively investigated in materials science and engineering due to their remarkable magnetic, electrical, and catalytic traits^7,8^. An exceptional example from the ferrite family is magnesium ferrite (MgFe₂O₄), which stands out for its robust magnetic properties and inverse spinel structure^9,10^. In this configuration, magnesium ions predominantly occupy octahedral sites, while iron ions are distributed across both tetrahedral and octahedral sites^11,12^. The unique architecture of MgFe₂O₄ allows it to fuse the advantageous magnetic features of both manganese and iron ferrites, making it highly versatile for applications such as data storage, magnetic hyperthermia therapy, catalysis, sensors, and electromagnetic wave absorption^13^. Heterogeneous magnetic catalysts represent a promising category within catalysis due to their distinctive combination of magnetic characteristics and catalytic efficacy^14,15^. These materials present significant advantages, including simplified catalyst recovery, enhanced recyclability, and improved reaction kinetics^16,17^. Compared to conventional catalytic systems, heterogeneous magnetic catalysts streamline the recovery process, cut costs, and reduce environmental footprints^18,19^. Their ability to be recycled reinforces their potential for long-term sustainability and economic practicality, positioning them as an appealing choice for a wide array of catalytic applications^20,21^. Furthermore, the active sites located on the surface of the magnetic core or functionalized coatings support a diverse range of catalytic reactions, broadening their applicability across multiple domains^11,16,18,22^. The Suzuki reaction has become a cornerstone in various scientific domains, particularly due to its versatility in forming carbon-carbon bonds^23–25^. This reaction is extensively utilized in pharmaceutical synthesis, agricultural chemistry, polymer production, advanced material development, and natural product chemistry^26,27^. Its ability to efficiently create bonds between diverse components makes it an indispensable tool in constructing intricate molecules^28,29^. In pharmaceuticals, the Suzuki reaction plays a crucial role in the creation of drug molecules and intermediates^30^. Many significant pharmaceutical compounds feature complex carbon-carbon bond frameworks, which can be assembled with relative ease using this method. Beyond pharmaceuticals, the reaction’s significance extends to materials science, where it aids in the development of conducting polymers, organic light-emitting diodes (OLEDs), organic solar cells, and nanoscale materials^31,32^. By enabling the polymerization of aryl and vinyl monomers often challenging to synthesize through other methods it facilitates precise control over polymer attributes such as chain length, molecular weight distribution, and overall structural design^33–35^. Its impact on organic chemistry is equally remarkable^36,37^. The synthesis of complex natural compounds, characterized by multi-ring structures and chiral centers, represents one of the field’s most demanding challenges^38–40^. The Suzuki reaction provides a streamlined approach by enabling the integration of large and intricate building blocks into these molecules, thereby enhancing synthetic efficiency while shortening reaction pathways^5,41^. Notably, this methodology has been instrumental in producing biologically active natural products like alkaloids, terpenes, and steroids, which hold immense significance in medical and industrial applications^42,43^.

The report details the development of an efficient heterogeneous catalyst, DT-CH-Pd, supported on MgFe_2_O_4_ magnetic nanoparticles and tailored for C-C coupling reactions. The findings demonstrate that the catalyst exhibits performance comparable to its homogeneous counterpart. Moreover, it provides the added benefit of easy recovery from the reaction mixture while maintaining nearly full catalytic activity.

## Experimental

### Preparation of MgFe_2_O_4_@APTES@DT-CH-Pd

**Fig. 1:**
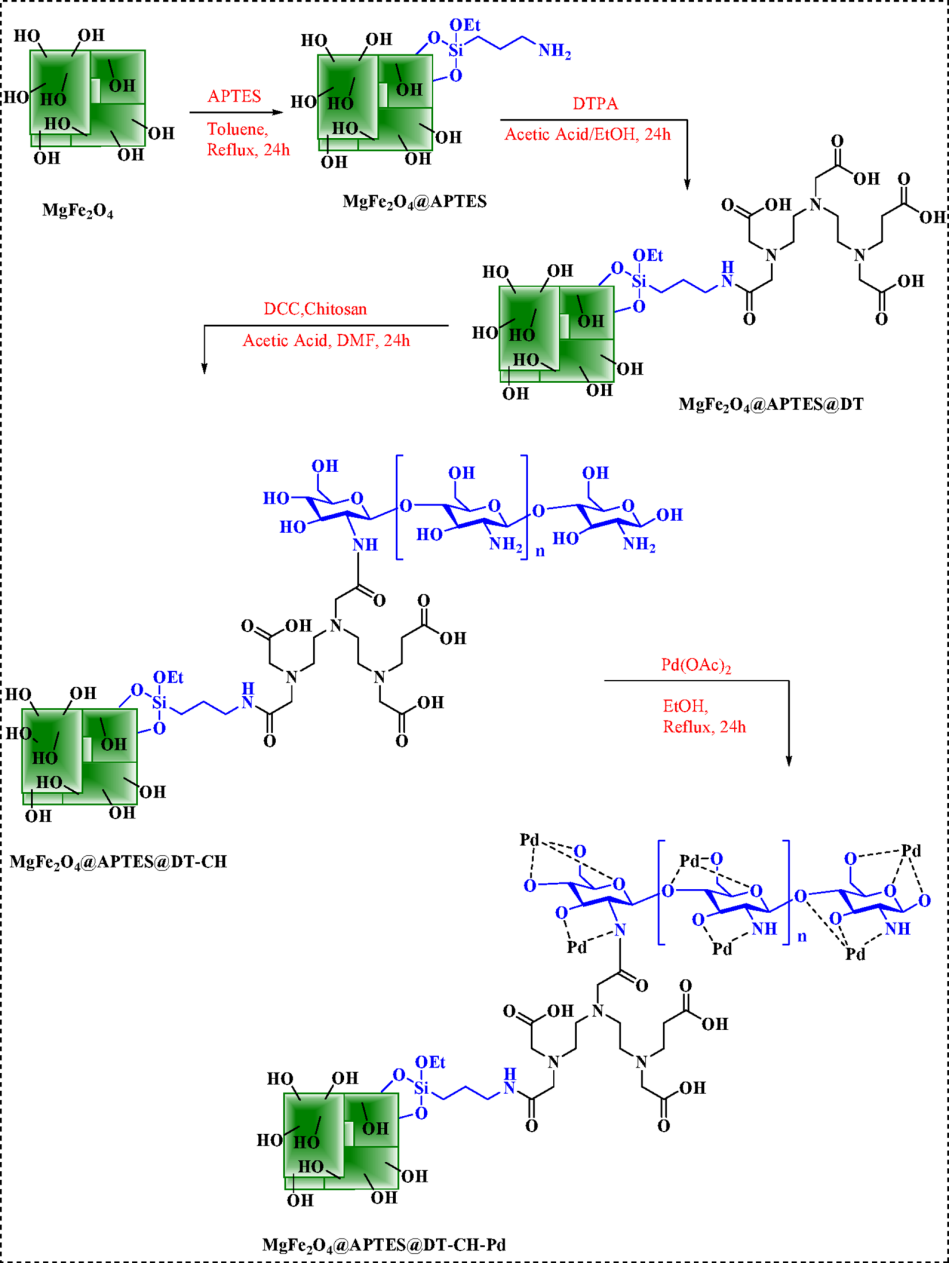
Synthesis of MgFe_2_O_4_@APTES@DT-CH-Pd.

The synthesis process for MgFe_2_O_4_ nanoparticles (NPs) began with the preparation of 3 mmol of Mg(NO_3_)_2_·6H_2_O and 6 mmol of FeCl_3_·6H_2_O. These components were combined and stirred in a water bath maintained at 80 °C for 30 min. Following this, 4 g of NaOH was introduced into the mixture, which was stirred continuously for 24 h. The resulting particles were separated using a straightforward magnetic method, thoroughly washed multiple times with water, and subsequently dried at 60 °C, as outlined in Fig. 1. To functionalize the material, 3.5 mL of 3-aminopropyltrimethoxysilane (APTES) was carefully introduced into a suspension containing 1 g of MgFe_2_O_4_ dispersed in 50 mL of toluene. This mixture was stirred under a nitrogen atmosphere at reflux conditions for 24 h to ensure thorough reaction. After the silanization process, the solid product was magnetically separated and dried at ambient temperature for 24 h before further modification. Subsequently, 1 g of nano-MgFe_2_O_4_@APTES was dispersed in a 1:1 ethanol-acetic acid solution (v/v) using an ultrasonic bath for 15 min. Afterwards, 2.5 mmol of diethylenetriaminepentaacetic acid (DTPA) was added to this dispersion, and the mixture was stirred at reflux for another 24 h. The resulting MgFe_2_O_4_@APTES@DT nanoparticles were recovered magnetically and sequentially washed with ethanol/acetic acid (1:1, v/v), water, and methanol. These multi-carboxyl-modified magnetic silica gels were then oven-dried at 60 °C for 12 h. For further functionalization, 1 g of MgFe_2_O_4_@APTES@DT was added to 30 mL of DMF and subjected to sonication for 15 min. Following this, 2 g of DCC was introduced, and 0.8 g of chitosan dissolved in a 25 mL solution of acetic acid (2.0% v/v) was gradually added to the system at room temperature. Upon complete chemical addition, the mixture was heated to 60 °C and maintained for 24 h. The resulting microgel was cooled to room temperature and thoroughly washed with deionized water. Next, 1 g of MgFe_2_O_4_@APTES@DT-CH was dispersed in ethanol, and 2.5 mmol of palladium acetate (Pd(OAc)_2_) was added. This mixture underwent reflux for 24 h, after which 0.5 g of sodium borohydride (NaBH_4_) was incorporated and allowed to react for an additional 3 h. The final nanoparticles, MgFe_2_O_4_@APTES@DT-CH-Pd, were isolated via magnetic decantation, rinsed with ethanol, and air-dried for further use as outlined in Fig. 1.

### Preparation of Suzuki reaction

**Fig. 2:**
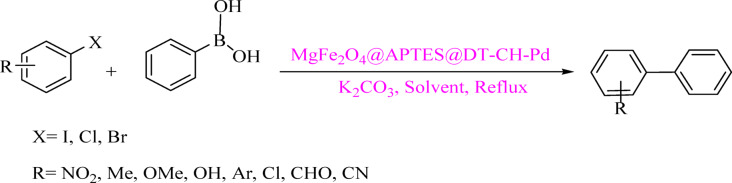
MgFe_2_O_4_@APTES@DT-CH-Pd catalyzed Suzuki coupling reactions.

Phenylboronic acid (1 mmol), an aryl halide (1 mmol), and K_2_CO_3_ (1.1 mmol) were added to 3 mL of water in the presence of MgFe_2_O_4_@APTES@DT-CH-Pd magnetic nanoparticles (0.02 g). The reaction mixture was stirred under reflux, with its progress monitored by thin-layer chromatography (TLC). Upon completion, the mixture was allowed to cool, and the magnetic nanoparticles were separated using an external magnet. These were then washed thoroughly with water and ethyl acetate. The remaining solution was subjected to liquid–liquid extraction using water and ethyl acetate. The organic phase was dried using 0.9 g of anhydrous sodium sulfate (K_2_SO_4_). After evaporating the ethyl acetate, pure biphenyl derivatives were obtained in excellent yields, as shown in Fig. 2.

## Selected NMR data

**1**,**1’-biphenyl**:^1^H NMR (400 MHz, DMSO) (Figure.S_1_): δ_H_ = 7.5 (m, 10 H), ppm.

**3-nitro-1**,**1’-biphenyl**:^1^H NMR (400 MHz, DMSO) (Figure.S_2_): δ_H_ = 8.2 (d, 2 H), 7.5 (m, 7 H), ppm.

**1**,**1’:4’**,**1’’-terphenyl**:^1^H NMR (400 MHz, DMSO) (Figure.S_3_): δ_H_ = 7.6 (s, 4 H), 7.3 (s, 4 H), 7.0 (m, 6 H), ppm.

**4-methyl-1**,**1’-biphenyl**:^1^H NMR (400 MHz, DMSO) (Figure.S_4_): δ_H_ = 7.8 (s, 1H), 6.6 (m, 2 H), 7.4 (d, 6 H), 1.0 (s, 3 H) ppm.

### Catalyst characterizations

**Fig. 3:**
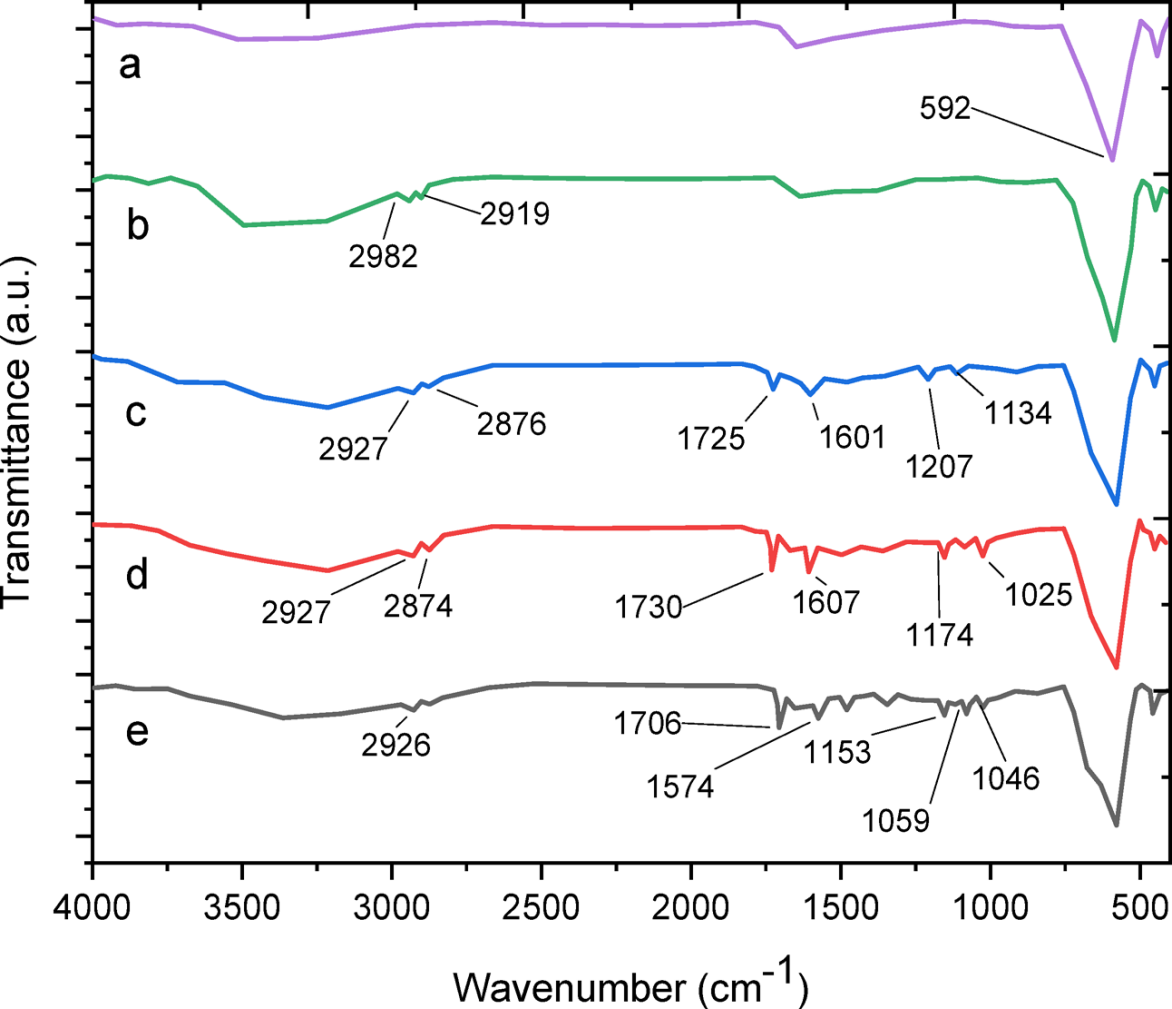
Comparative study of FTIR spectra of (a) MgFe_2_O_4_, (b) MgFe_2_O_4_@APTES, (c) MgFe_2_O_4_@APTES@DT, (d) MgFe_2_O_4_@APTES@DT-CH, (e) MgFe_2_O_4_@APTES@DT-CH-Pd.

To investigate the surface properties of composite nanoparticles, their molecular structure was analyzed using FT-IR spectra (Fig. 3). The characteristic absorption peak at 592 cm⁻¹ for MgFe₂O₄ corresponds to the Fe–O bond’s stretching vibration. This peak was consistently observed across all spectra, confirming the presence of MgFe₂O₄ nanoparticles in the intermediates and final products (Fig. 3a). Following functionalization with APTMS, new absorption peaks at 2985 and 2919 cm⁻¹ emerged, representing the symmetric vibrations of aliphatic C–H stretching within the methylene group of the silane coupling agent. This indicates successful grafting of amino groups onto the MgFe₂O₄ surface (Fig. 3b). Subsequent grafting of multi-carboxyl groups introduced strong absorption bands at 1725 and 1207 cm⁻¹, attributed to the C = O and C–O stretching vibrations of the –COO– group, confirming the formation of carboxylic groups (DT) on the nanoparticle surface (Fig. 3c). Chitosan was then grafted onto the magnesium ferrite surface. While the N–H stretching band at 3400 cm⁻¹ overlapped with hydroxy group bands, the N–H bending mode at 1025 cm⁻¹, along with evidence of C–O–C groups, confirmed the presence of chitosan. Additionally, C–H stretching vibrations at 2927 and 2874 cm⁻¹ indicated aliphatic CH groups in chitosan (Fig. 3d). Finally, due to significant palladium content on the nanoparticle surface, reductions in C = O and C–O vibrations suggest that the carboxyl groups on the magnetic silica surface functionally coordinate palladium, verifying the successful immobilization of palladium complexes on the MgFe₂O₄@APTES@DT-CH surface (Fig. 3e.)^44–48^.

**Fig. 4:**
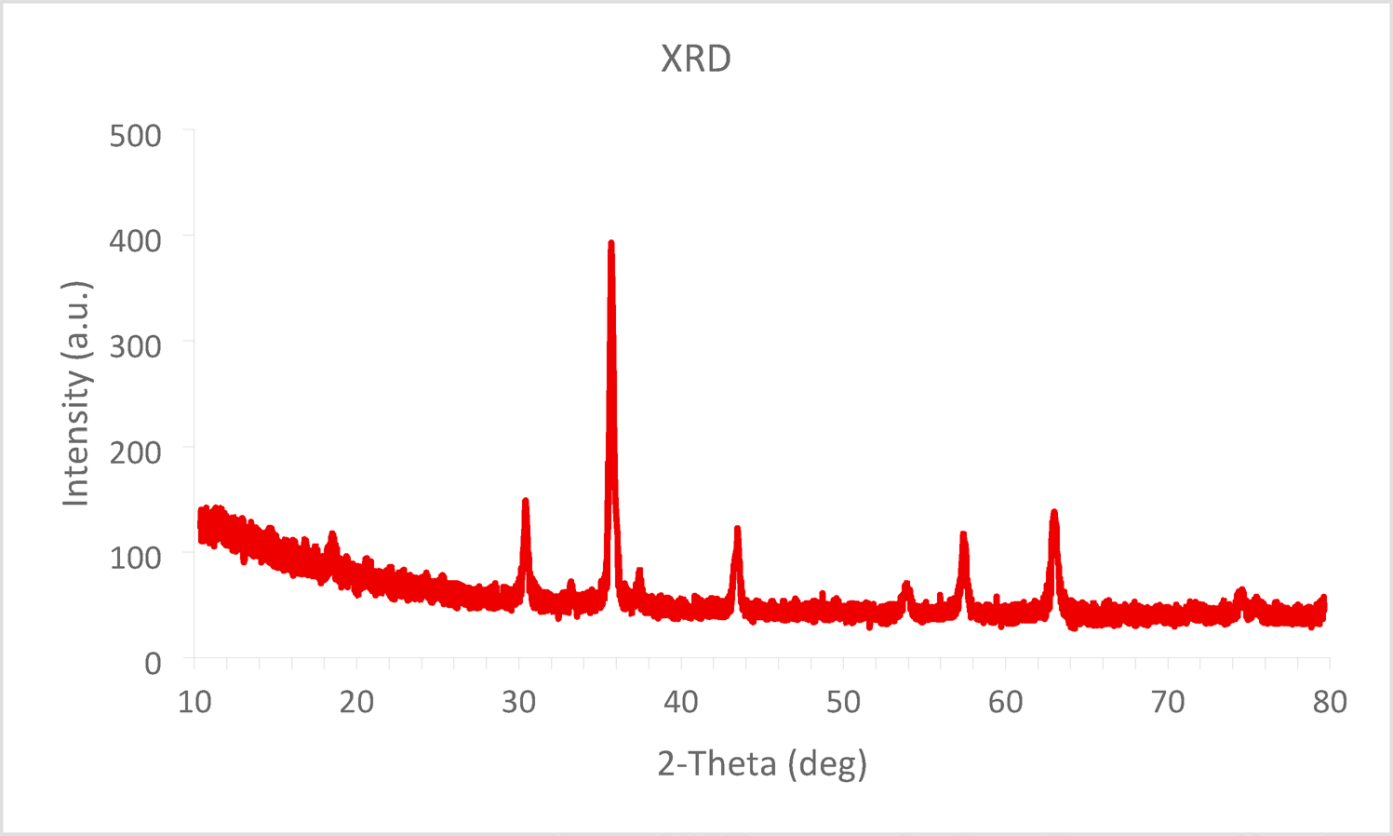
XRD spectrum of MgFe_2_O_4_@APTES@DT-CH-Pd.

The X-ray diffraction (XRD) analysis of the MgFe_2_O_4_@APTES@DT-CH-Pd nanocomposite, as shown in Fig. 4, reveals distinct diffraction peaks at 2θ angles of 30°, 35°, 43°, 53°, 57°, and 63°. These peaks are associated with the (220), (311), (400), (422), (511), and (440) crystal planes, indicative of the cubic spinel structure characteristic of MgFe2O4. This confirms the successful synthesis of MgFe_2_O_4_ nanoparticles. Importantly, the crystal structure of MgFe_2_O_4_ remained preserved even after surface modification with various organic functional groups. Additionally, using the Debye–Scherrer formula, the mean crystal size of MgFe_2_O_4_@APTES@DT-CH-Pd MNPs was calculated to be 16.69 nm^49,50^.

**Fig. 5:**
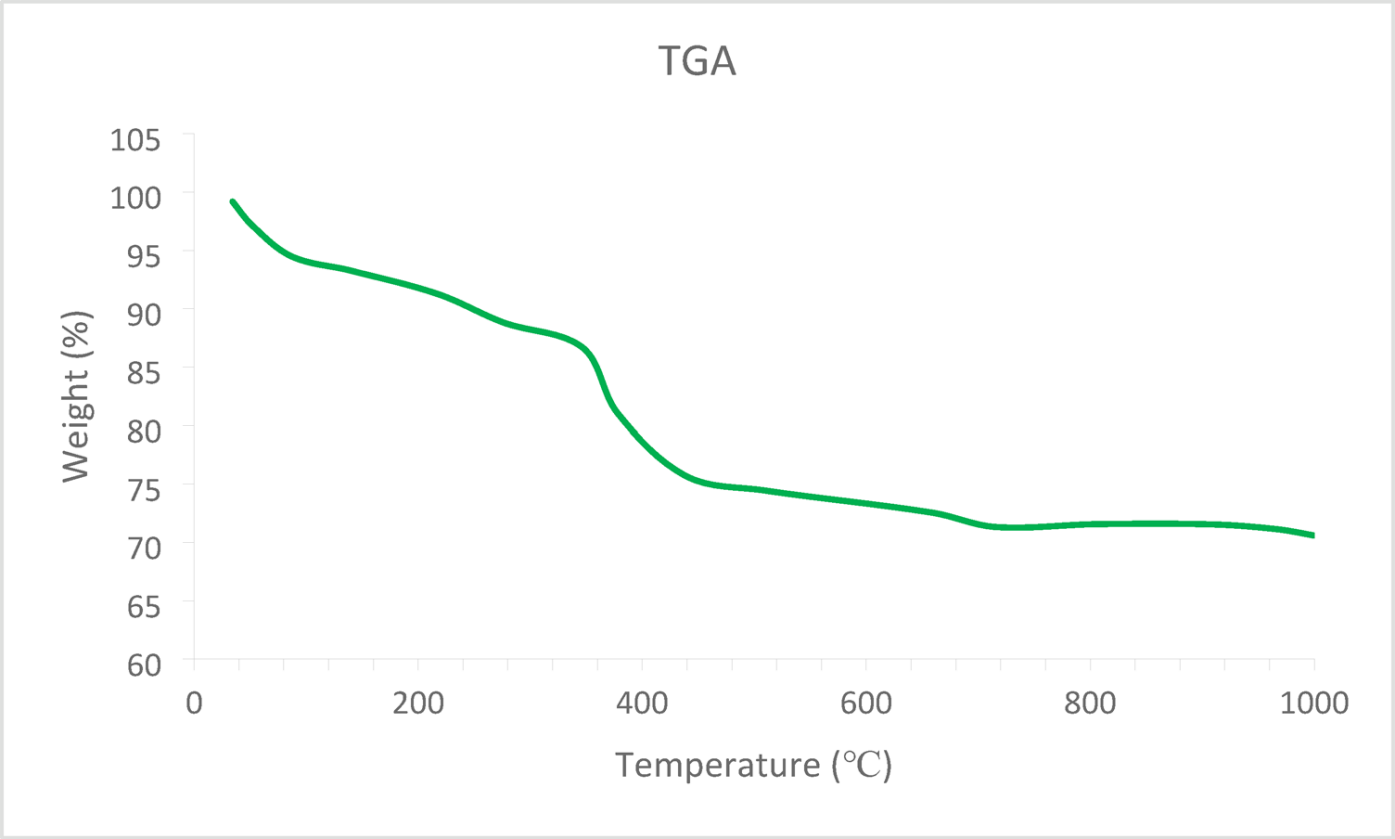
TGA curve of MgFe_2_O_4_@APTES@DT-CH-Pd.

The thermal behavior of the MgFe₂O₄@APTES@DT-CH-Pd nanocomposite was evaluated using thermogravimetric analysis (TGA), as depicted in Fig. 5. The analysis identified two distinct stages of weight loss. The first stage, occurring below 250 °C, was linked to the evaporation of the adsorbed solvent. The second stage, noted between 250 and 700 °C, revealed a prominent weight loss of approximately 15%, which was attributed to the thermal decomposition of the organic layer and the palladium (Pd) complex bonded to it. These results emphasize the existence of a strong chemical bond between the DT-CH-Pd complex and the surface of the MgFe₂O₄ magnetic nanoparticles.

**Fig. 6:**
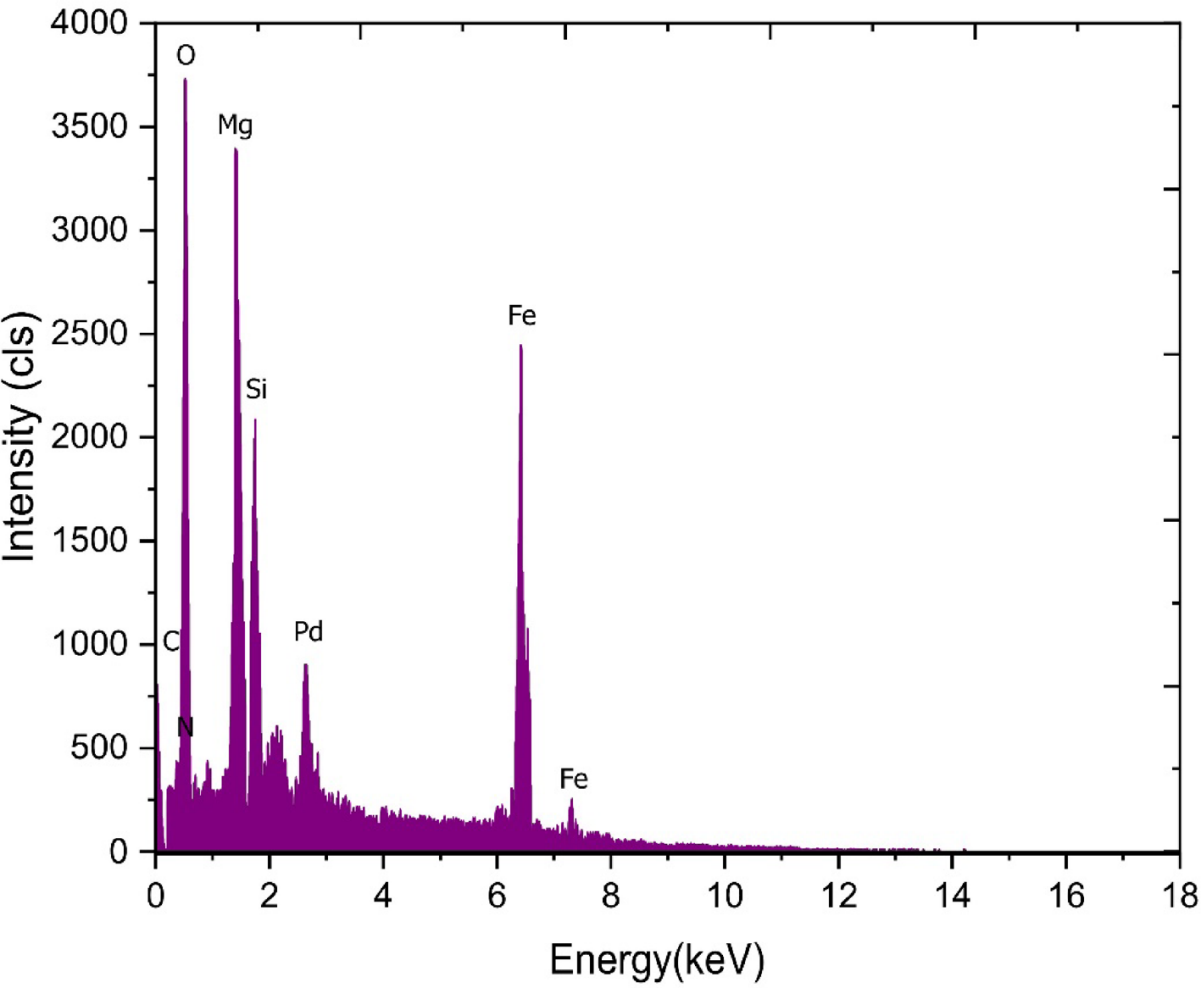
EDS analysis of MgFe_2_O_4_@APTES@DT-CH-Pd.

The elemental composition of MgFe₂O₄@APTES@DT-CH-Pd was assessed using an EDX spectrum, as depicted in Fig. 6. This analysis confirmed the presence of nitrogen, oxygen, iron, carbon, magnesium, silicon, and palladium within the catalyst, demonstrating the successful synthesis of the nanoparticles. Furthermore, it verified the effective immobilization of DT-CH-Pd onto the surface of the MgFe₂O₄ magnetic nanoparticles. To measure the palladium content in MgFe₂O₄@APTES/CC/GA-Pd, ICP-OES analysis indicated a Pd concentration of 1.7 × 10⁻³ mol g⁻¹. In addition, ICP analysis was conducted to evaluate palladium leaching after the catalyst was recycled. The results showed a Pd concentration of 1.6 × 10⁻³ mol g⁻¹ in the reused catalysts, suggesting minimal Pd leaching from the MgFe₂O₄@APTES@DT-CH-Pd structure.

**Fig. 7:**
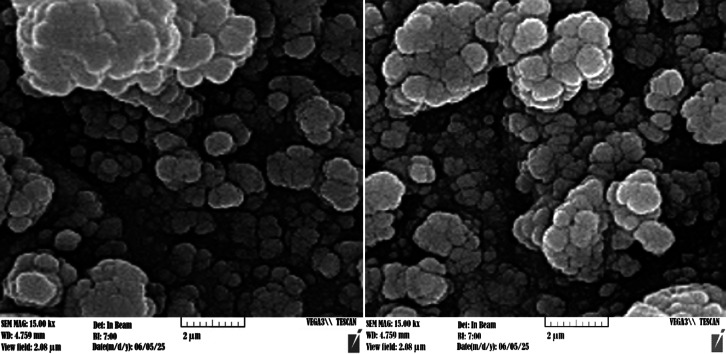
SEM images of MgFe_2_O_4_@APTES@DT-CH-Pd.

Scanning electron microscopy (SEM) was employed to assess and precisely characterize the size and structural attributes of the MgFe_2_O_4_@APTES@DT-CH-Pd nanocomposite. The resulting images, displayed in Fig. 7, reveal spherical particles with nanoscale dimensions, verifying the successful synthesis of nanoparticles with meticulous dimensional control.

**Fig. 8:**
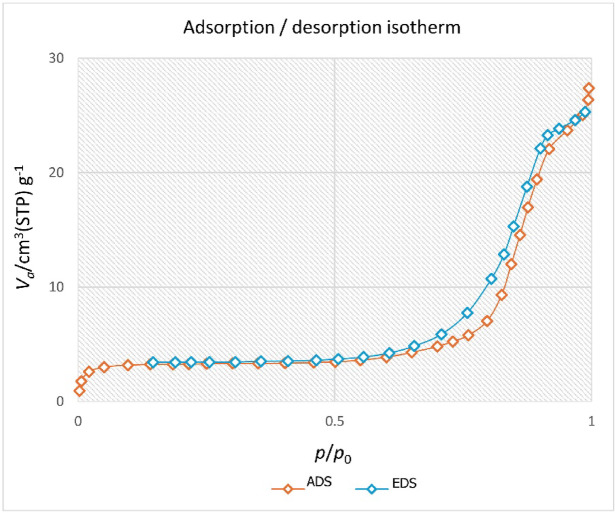
The N_2_ adsorption–desorption isotherm of MgFe_2_O_4_@APTES@DT-CH-Pd complex.

The MgFe_2_O_4_@APTES@DT-CH-Pd complex was investigated using BET analysis to assess its isotherm type, surface area, and pore features, as depicted in Fig. 8. The analysis yielded values of 28.4 nm for average pore diameter, 41.36 m²/g for specific surface area, and 0.062 cm³/g for total pore volume. The nanocomposite demonstrated a type IV isotherm and revealed a mesoporous structure, as indicated by its pore characteristics. These surface attributes of the MgFe_2_O_4_@APTES@DT-CH-Pd complex offer abundant active sites for direct interactions with organic reactants, contributing to enhanced catalytic performance.

**Fig. 9:**
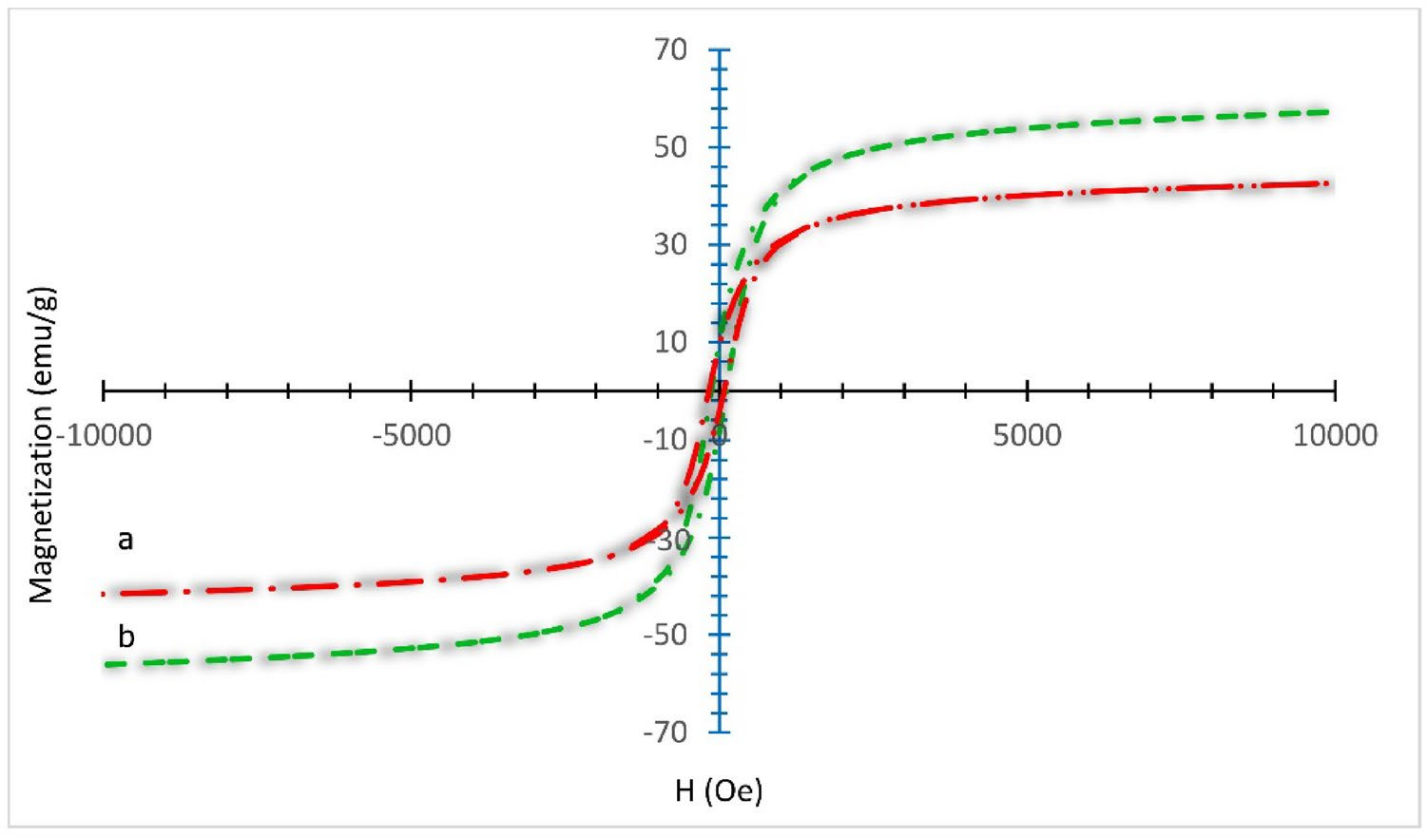
VSM curves of (a) MgFe_2_O_4_ (b) MgFe_2_O_4_@APTES@DT-CH-Pd.

To assess the magnetic properties, uncoated magnetic spinel ferrite (MgFe_2_O_4_) and the MgFe_2_O_4_@APTES@DT-CH-Pd nanocomposite (MNPs) were analyzed using a vibrating sample magnetometer (VSM) at room temperature under an external magnetic field range of ± 10,000 Oe, as depicted in Fig. 9. The VSM analysis revealed a reduction in saturation magnetization (Ms), from approximately 53 emu/g for MgFe_2_O_4_ to around 32 emu/g for MgFe_2_O_4_@APTES@DT-CH-Pd. This decline in Ms is attributed to the presence of the newly applied coating layer, confirming the successful synthesis of the modified catalyst. Despite this lower magnetization, the MgFe_2_O_4_@APTES@DT-CH-Pd exhibits sufficient magnetic susceptibility to enable efficient magnetic separation across various reaction environments.

### Catalytic studies

Following the successful synthesis and characterization of MgFe_2_O_4_@APTES@DT-CH-Pd magnetic nanoparticles, their catalytic efficiency in the Suzuki reaction was assessed. A model reaction was conducted, coupling iodobenzene (1 mmol) with phenylboronic acid (1 mmol), to identify the ideal reaction parameters. Various factors such as catalyst quantity, solvent choice, type of base, and reaction temperature were systematically examined, with the results summarized in Table 1. Initially, the impact of catalyst concentration was investigated, revealing that the highest yield was achieved using 0.02 g of MgFe_2_O_4_@APTES@DT-CH-Pd nanoparticles; notably, no reaction occurred in the absence of a catalyst. Further tests identified water as the optimal solvent and K_2_CO_3_ as the most effective base for the carbon-carbon coupling product under reflux conditions. Ultimately, the optimal parameters determined were 0.02 g of catalyst, 1.2 mmol of K_2_CO_3_, and water under reflux conditions.

**Table 1:** Optimizing the reaction between Phenylboronic acid and Iodobenzene using MgFe_2_O_4_@APTES@DT-CH-Pd as the catalytic system.


Entry	Cat. (g)	Solvent	Base	Temperature (°C)	Time (min)	Yield (%)
1	-	water	K_2_CO_3_	Reflux	24 h	-
2	0.008	water	K_2_CO_3_	Reflux	30	45
3	0.01	water	K_2_CO_3_	Reflux	30	60
4	0.02	water	K_2_CO_3_	Reflux	30	98
5	0.03	water	K_2_CO_3_	Reflux	30	98
6	0.02	PEG-400	K_2_CO_3_	120	30	54
7	0.02	Solvent Free	K_2_CO_3_	90	30	62
8	0.02	DMSO	K_2_CO_3_	100	30	75
9	0.02	EtOH	K_2_CO_3_	Reflux	30	70
10	0.02	DMF	K_2_CO_3_	100	30	63
11	0.02	water	KOH	Reflux	30	49
12	0.02	water	NaOH	Reflux	30	56
13	0.02	water	t-BuOK	Reflux	30	88
14	0.02	water	Na_2_CO_3_	Reflux	30	43
15	0.02	water	Et_3_N	Reflux	30	39

Following optimization of the reaction conditions, the MgFe_2_O_4_@APTES@DT-CH-Pd (MNPs) magnetic nanoparticle catalyst was tested in Suzuki C-C cross-coupling reactions across a diverse range of aryl halides and phenylboronic acid. The catalyst demonstrated moderate to excellent efficiency in yielding biphenyl derivatives, effectively accommodating various aryl halides such as iodides, bromides, and chlorides with both electron-donating and electron-withdrawing substituents. Reactions involving aryl halides with electron-donating groups required longer times, likely due to their reduced activation during the oxidative addition step compared to electron-withdrawing substituents. The electrophilic reactivity pattern adhered to the order I > Br > Cl, with chlorides showing the least reactivity. However, the presence of DT-CH as a ligand alongside K_2_CO_3_ as a base enabled successful coupling of chlorides. In experiments utilizing 1-bromo-4-chlorobenzene, the bromide group exhibited higher reactivity than the chloride group, presenting a significant advantage for further functionalization of the resulting products (Table 2).

**Table 2:** Synthesis of carbon-carbon coupling reactions from Aryl halides utilizing MgFe_2_O_4_@APTES@DT-CH-Pd.


Entry	Aryl halide	Product	Time (min)	Yield (%)	TON	TOF (min^− 1^)
1			30	98	28.8	57.6
2			60	92	27.0	27.0
3			30	94	27.6	55.2
4			45	95	27.9	37.2
5			30	93	27.3	54.6
6			30	96	28.2	56.4
7			120	81	23.8	11.9
8			60	90	26.4	26.4
9			60	90	26.4	26.4
10			45	92	27.0	36
11			30	94	27.6	55.2
12			45	90	26.4	35.2
13			60	94	27.6	27.6
14			30	91	26.7	53.4

**Fig. 10:**
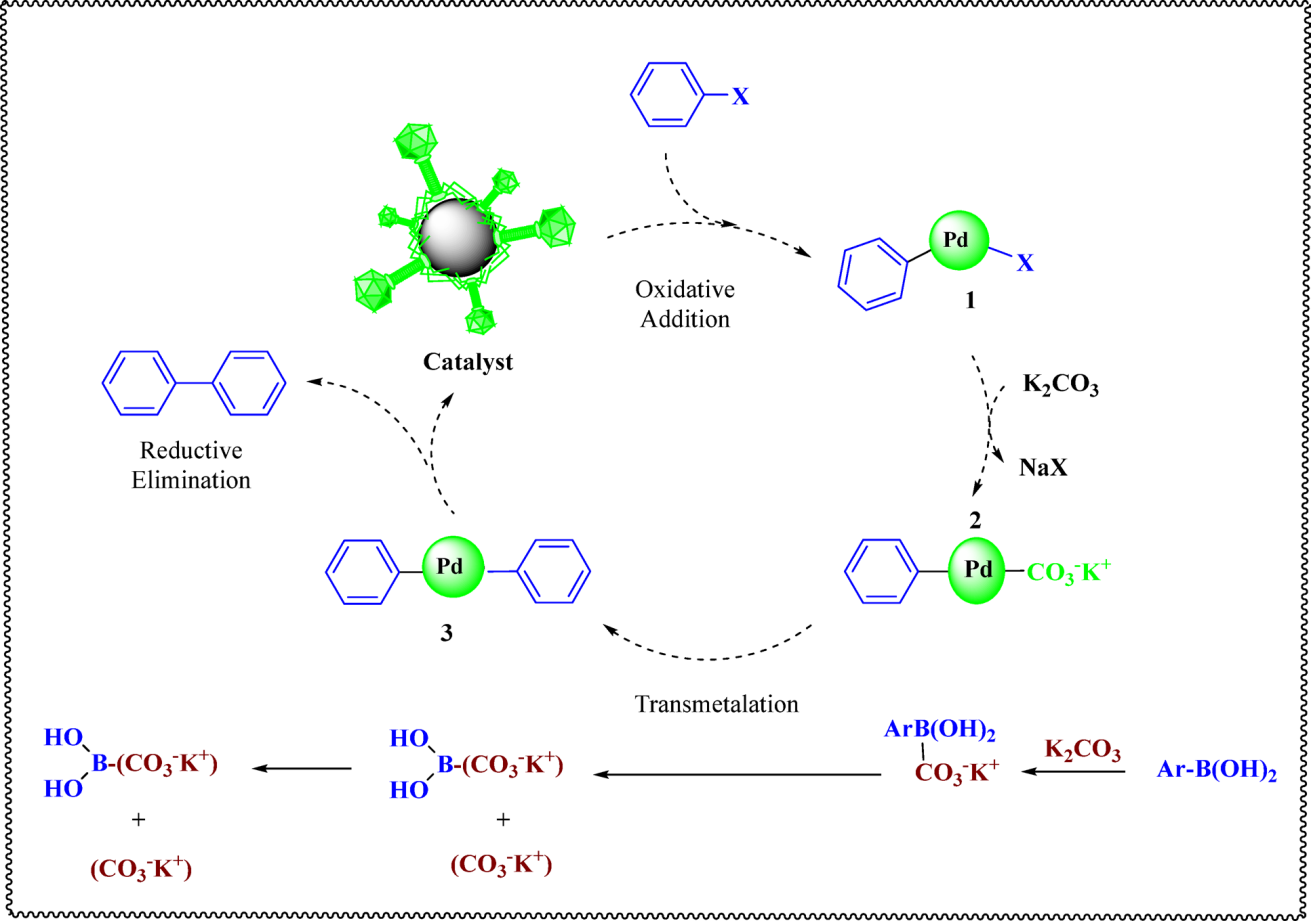
Possible mechanism for Suzuki reaction.

The catalytic cyclic mechanism of the Suzuki reaction employing the MgFe_2_O_4_@APTES@DT-CH-Pd catalyst follows three essential steps: oxidative addition, transmetalation, and reductive elimination. The process begins with the aryl halide interacting with the catalyst complex through oxidative addition, resulting in the formation of intermediate (1) This step is considered the most challenging phase of the cycle, where electron-donating oxygen groups present in the DT-CH ligand facilitate the cleavage of the Ar-X bond, effectively activating the Pd catalyst. Subsequently, intermediate 1 undergoes a transmetalation reaction involving the organoborane reagent and base, leading to the creation of intermediate (2) In the final stage, reductive elimination occurs, yielding the desired product while regenerating the MgFe_2_O_4_@APTES@DT-CH-Pd catalyst, which is then ready to participate in further catalytic cycles (Fig. 10).

### Catalyst leaching study

The stability and heterogeneity of the MgFe_2_O_4_@APTES@DT-CH-Pd catalyst in the Suzuki reaction, specifically the coupling of iodobenzene and phenylboronic acid, were evaluated through a hot filtration experiment conducted midway through the reaction. During the initial test, the product yield at this stage reached 55%. In a subsequent experiment where the catalyst was removed at the same point, the reaction proceeded slightly further, resulting in a small increase in yield to 57%, after which no additional progress was noted. These observations suggest that at least palladium is leaching from the catalyst during the reaction.

## Catalyst recyclability

**Fig. 11:**
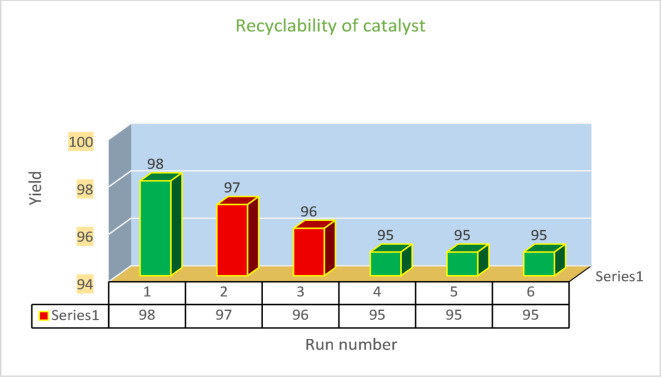
Recyclability of MgFe_2_O_4_@APTES@DT-CH-Pd.

Recovering and reusing nanocatalysts represent a critical feature in catalytic processes. To evaluate this functionality, we studied the performance of the recycled catalyst in the model reaction under optimized conditions. The findings revealed that the reused catalyst retained consistent efficiency, successfully enabling biphenyl synthesis across six cycles without noticeable loss in its initial catalytic activity, as illustrated in Fig. 11. The initial decrease from 98% to 97%, then to 96%, and finally to 95%, suggests that the deactivation mechanisms are more active in the early cycles and then reach a more stable state. This may be due to the faster loss of the “more vulnerable active sites” in the first cycles, reaching an equilibrium between degradation and catalyst stability in the later cycles.

The efficiency of MgFe_2_O_4_@APTES@DT-CH-Pd as a nanocatalyst was evaluated by comparing its performance with that of other nanocatalysts reported in the literature, as summarized in Table 3. This comparison underscores its effectiveness in C-C coupling reactions relative to previously documented catalysts. Importantly, the nano-MgFe_2_O_4_@APTES@DT-CH-Pd exhibits significant advantages, such as reduced reaction times, excellent product yields, and the use of environmentally benign solvents. These results highlight its potential as a highly effective and versatile nanocatalyst for the synthesis of organic compounds.

**Table 3:** Comparison of the catalytic activity of MgFe_2_O_4_@APTES@DT-CH-Pd with previously documented methods in the Suzuki reaction.

Entry	Catalyst	Ar-X	Product	Time (min)	Yield (%)	Ref.
1	MCM-Pd			1440	74	^51^
2	MCM-41-VO			720	100	^52^
3	MCM@ACD-Pd			120	94	^53^
4	MgFe_2_O_4_@APTES@DT-CH-Pd			30 min	98	**This work**

## Conclusion

The study introduces MgFe_2_O_4_@APTES@DT-CH-Pd, an innovative metallic nanocatalyst successfully developed, synthesized, and thoroughly characterized using cutting-edge methods such as FT-IR, TGA, EDS, SEM, XRD, ICP, and VSM. This catalyst demonstrated remarkable efficiency in facilitating C-C coupling reactions, delivering high yields within short timeframes. The functionalization of MgFe_2_O_4_ nanoparticles with DT-CH-Pd significantly boosted their catalytic activity and overall reaction performance. Furthermore, it exhibited excellent reusability by maintaining steady efficiency across multiple usage cycles. Importantly, the MgFe_2_O_4_@APTES@DT-CH-Pd nano catalyst is easily synthesized from cheap and available materials and has several advantages, including easy separation, thermal stability in the reaction mixture, and reusability.

## Supplementary Information

Below is the link to the electronic supplementary material.

Supplementary Material 1

## Data Availability

All data generated or analyzed during this study are included in this published article and its supplementary information files.
