# Supplementary material for: Palladium nanoparticles immobilized on DT-CH-modified MgFe2O4@APTES magnetic nanoparticles as an efficient and reusable new catalyst for C-C coupling reactions
Source: Sci Rep. 2025 Nov 25;15:41928. doi: 10.1038/s41598-025-25753-3 (PMC12647865; doi:10.1038/s41598-025-25753-3)
Supplement: Supplementary file 1 — Supplementary Material 1 [file 41598_2025_25753_MOESM1_ESM.docx]

**Palladium nanoparticles immobilized on DT-CH‐modified MgFe_2_O_4_@APTES magnetic nanoparticles as an efficient and reusable new catalyst for C-C coupling reactions**


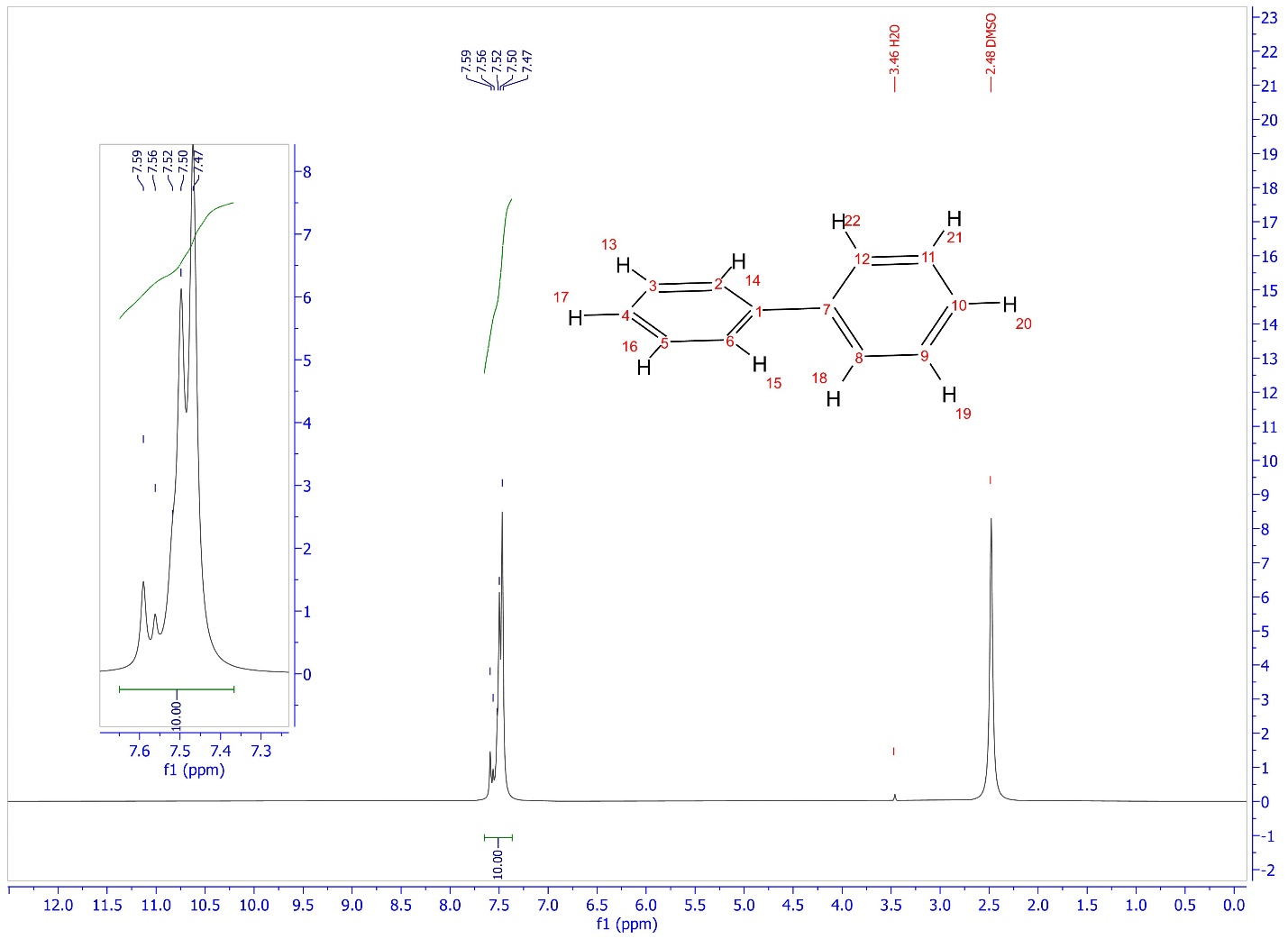


Figure.S_1_. 1,1'-biphenyl

**1,1'-biphenyl**:^1^H NMR (400 MHz, DMSO): δ_H_= 7.5 (m, 10H), ppm.


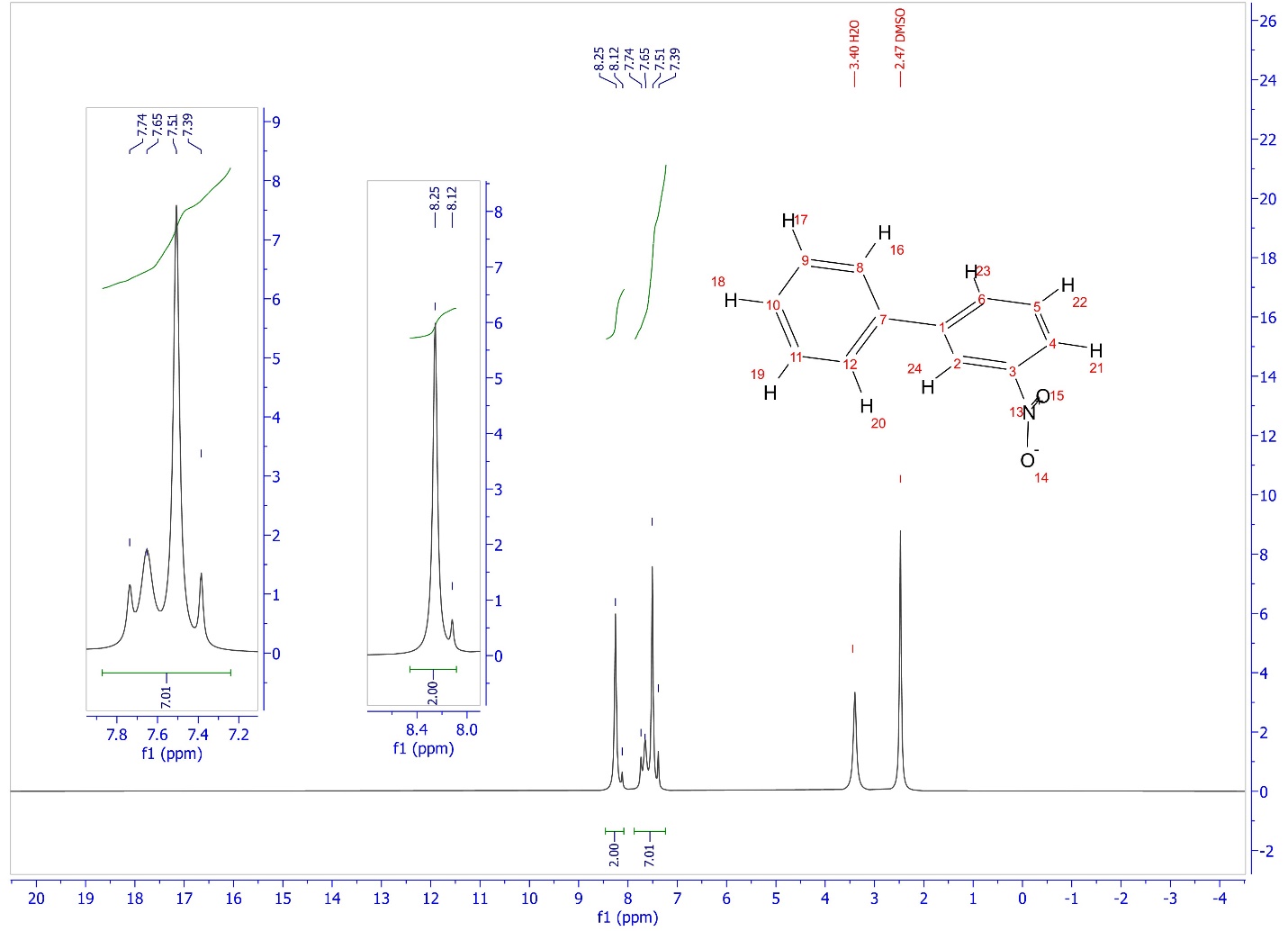


Figure.S_2_. 3-nitro-1,1'-biphenyl

**3-nitro-1,1'-biphenyl**:^1^H NMR (400 MHz, DMSO): δ_H_= 8.2 (d, 2H), 7.5 (m, 7H), ppm.


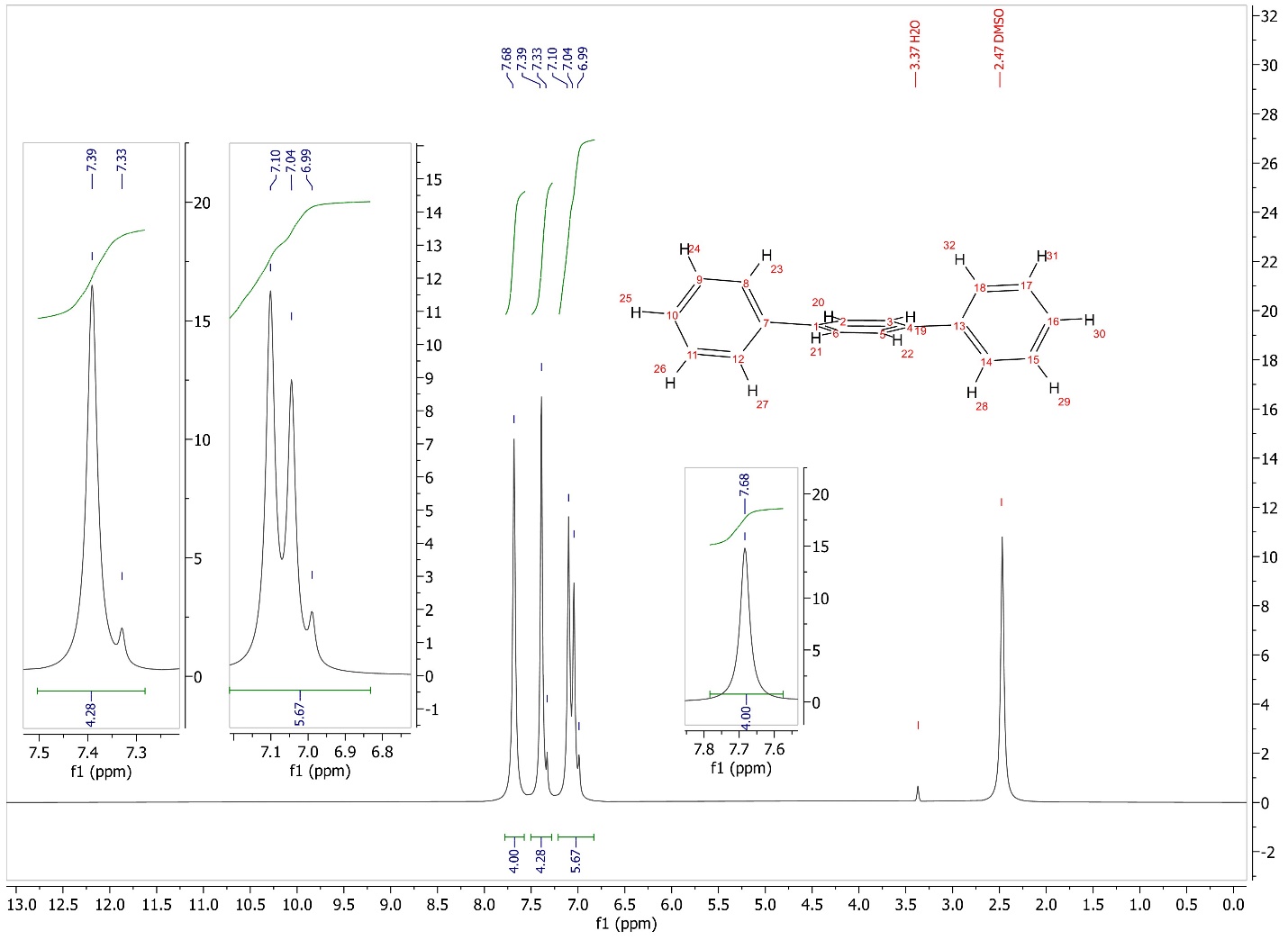


Figure.S_3_. 1,1':4',1''-terphenyl

**1,1':4',1''-terphenyl**:^1^H NMR (400 MHz, DMSO): δ_H_= 7.6 (s, 4H), 7.3 (s, 4H), 7.0 (m, 6H), ppm.


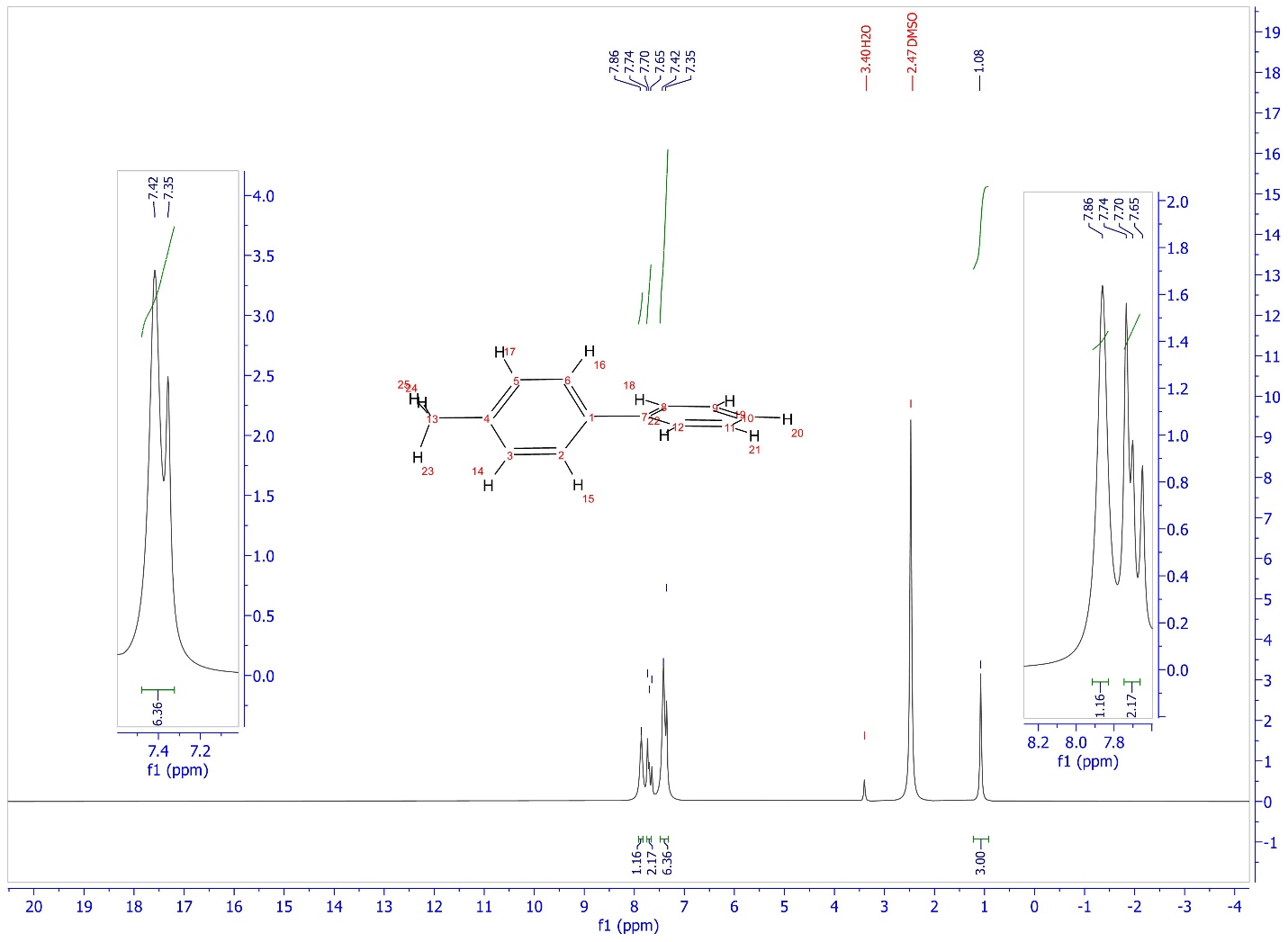


Figure.S_4_. 4-methyl-1,1'-biphenyl

**4-methyl-1,1'-biphenyl**:^1^H NMR (400 MHz, DMSO): δ_H_= 7.8 (s, 1H), 6.6 (m, 2H), 7.4 (d, 6H), 1.0 (s, 3H) ppm.
